# Harmonizing Prokaryotic Nomenclature: Fixing the Fuss over Phylum Name Flipping

**DOI:** 10.1128/mbio.00970-22

**Published:** 2022-05-10

**Authors:** Adyasha Panda, Salim T. Islam, Gaurav Sharma

**Affiliations:** a Institute of Bioinformatics and Applied Biotechnology (IBAB), Bengaluru, Karnataka, India; b Institut National de la Recherche Scientifique (INRS), Centre Armand-Frappier Santé Biotechnologie, Université du Québec, Institut Pasteur International Network, Laval, Quebec, Canada; c PROTEO, the Quebec Network for Research on Protein Function, Engineering, and Applications, Université Laval, Quebec, Quebec, Canada; The Ohio State University

**Keywords:** taxonomy, nomenclature, systematics, classification, *Bacteria*, *Archaea*, International Code of Nomenclature of Prokaryotes (ICNP), polyphasic taxonomy

## Abstract

Lloyd and Tahon recently criticized proposed bacterial phylum nomenclature changes (K.G. Lloyd, G. Tahon, Nat Rev Microbiol 20:123-124, 2022, https://doi.org/10.1038/s41579-022-00684-2) precipitated by the International Committee on Systematics of Prokaryotes (ICSP)’s official recognition of phylum nomenclature rules. Here, we extend the critique. While we applaud bringing consistency to phylum names, we prognosticate what this minute but momentous change entails for the future of microbial nomenclature and how this will sow confusion among researchers. Several pitfalls of the proposed ICSP framework-based nomenclature are also detailed, including (i) improper type genus name and suffix usage, (ii) loss of Bacteria/Archaea distinctions, (iii) disruption of major phylum name prefixes, and (iv) absence of organism name prevalidation. Finally, we suggest new names for the key bacterial phyla Proteobacteria (*Proteobacteriota*), Firmicutes (*Firmicuteota*), Actinobacteria (*Actinobacteriota*), and Tenericutes (*Tenericuteota*), while keeping the archaeal phylum names Crenarchaeota, Thaumarchaeota, and Euryarchaeota. Together, these changes will help researchers attain chaos-free uniform nomenclature.

## OPINION/HYPOTHESIS

Taxonomy encompasses both nomenclature and classification, which cumulatively contribute to a hierarchical organization of organisms based on their shared properties. It thus aids immensely in the effective communication and discussion of organismal diversity by researchers worldwide (1, 2). While hardly a matter of everyday concern, bacterial nomenclature affects the ways in which microbiology is described, taught, and perceived by the community. It is firmly established that polyphasic taxonomy—based on physiological, morphological, and genetic characterizations—and modern genome sequencing can serve as robust benchmarks for effective microbial classification, uncovering phylogenetic novelty at an unprecedented pace (3). Careful general considerations, principles, nomenclature rules with recommendations, and advisory notes exist in the International Code of Nomenclature of Prokaryotes (ICNP) set out by the International Committee on Systematics of Prokaryotes (ICSP) regarding the naming of bacterial class, order, family, genus, and species ranks (4). However, the rank of phylum had been historically overlooked until members of the ICSP recently voted to amend the ICNP to enshrine “phylum” under official nomenclature rules (5). Oren and Garrity subsequently proposed name changes for all 42 recognized prokaryotic phyla (6) using the suggested ICNP framework of (i) specifying the root word based on the type genus and (ii) adding a constant/uniform suffix (see Table S1 in the supplemental material).

10.1128/mbio.00970-22.1 TABLE S1 Representation of phylum names, respective type genera, and importance based on PubMed publications and sequenced genomes. The green-highlighted text represents the established phylum names. The yellow-highlighted text represents those phyla that previously belonged to a class rank or an order and have now been reclassified as phyla. Gray-highlighted text represents phyla for which the names have not been changed. The bold red text represents those phyla where a different suffix (besides “-ota”) has been added to the type genus name. Download Table S1, XLSX file, 0.01 MB. Copyright © 2022 Panda et al. 2022 Panda et al. https://creativecommons.org/licenses/by/4.0/ This content is distributed under the terms of the Creative Commons Attribution 4.0 International license.

The recognition of “phylum” under the ICNP is laudable, especially given the Wild West-like lack of rules prior to this decision. Under the proclaimed changes, “-ota” is appended as a suffix to all phylum names to achieve uniformity, a decision we applaud. Furthermore, with the new emphasis on using a root word based on the type genus name, revised names for several existing phyla will not be troublesome, as the name changes do not significantly diverge from the established names, i.e., Bacteroidetes (proposed: *Bacteroidota*), Chlamydiae (proposed: *Chlamydiota*), Spirochaetes (proposed: *Spirochaetota*), etc. However, in key instances, proposed changes have led to drastically different phylum names for several widely studied and long-recognized phyla, such as Proteobacteria (proposed: *Pseudomonadota*), Firmicutes (proposed: *Bacillota*), Actinobacteria (proposed: *Actinomycetota*), *Tenericutes* (proposed: Mycoplasmatota), Crenarchaeota (proposed: *Thermoproteota*), and Thaumarchaeota (proposed: *Nitrososphaerota*) (6) (Table S1). In addition, although the ICSP does not govern classification, some monophyletic groups have recently been reclassified and renamed from their former class/order names, including (i) order Bdellovibrionales to phylum *Bdellovibrionota*, (ii) class Epsilonproteobacteria to phylum *Campylobacterota*, and (iii) order Myxococcales (*Myxobacteria*) to phylum *Myxococcota* (7). These suggested changes have already been incorporated by the Genome Taxonomy Database (GTDB), which uses the relative evolutionary divergence (RED) metric (based exclusively on genome similarity score) instead of polyphasic taxonomy to establish taxonomic ranks (8).

**FIG 1:**
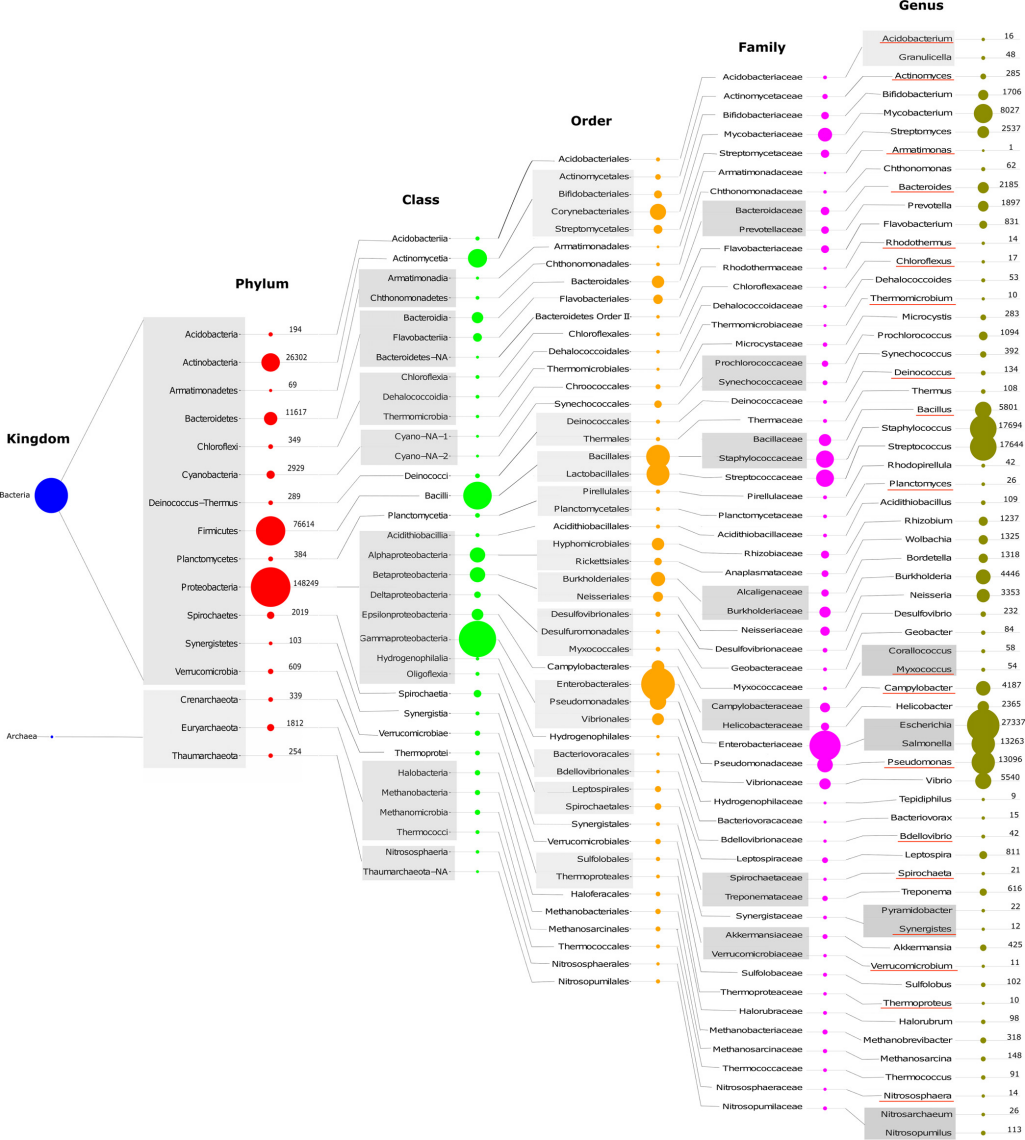
Distribution of sequenced genomes per taxa according to NCBI Taxonomy. Data (prokaryotes.txt) used in this analysis was downloaded from NCBI FTP page (https://ftp.ncbi.nlm.nih.gov/genomes/GENOME_REPORTS/) on 20 January 2022. Each red-underlined genus name is the well-known type genus in its respective phylum.

In response (9) to Lloyd and Tahon (10), within the context of the implementation of its pronounced changes to phylum naming conventions, the ICSP states that “…replacements for some commonly used colloquial names may cause some short-term displeasure or misunderstanding but also emphasize that this will be offset by the clear long-term benefit to the research community.” This is an entirely flippant viewpoint espoused by the ICSP. The significance of the phyla *Proteobacteria*, *Firmicutes*, *Actinobacteria*, *Tenericutes*, *Crenarchaeota*, and *Thaumarchaeota* to the body of microbial knowledge accumulated over a century of prokaryote systematics (11) cannot be overstated. These six phyla appear in 89% of PubMed entries (not including books) and 91% of sequenced genome records available in the NCBI Genome database; these numbers represent the cumulative search results of these six phyla out of the 42 phyla in the respective databases (Fig. 1).

Phylum names for organisms, genome sequences, respective taxonomy, and relevant information in other databases (such as SILVA, NR, UniProt, Swiss-Prot, PDB, etc.) can be easily modified; however, such alterations are impossible in published research articles, books, and educational material from across the globe in the last century. Changing long-established phylum names to entirely different ones in databases, but not in previous publications, will needlessly create chaos in scientific analyses as well as in the reading, referencing, and comparison of past versus future microbiology articles, open-access genomics, and metagenomic data sets. The ICSP response (9) also states that “…once names are proposed the community still decides which to adopt, although experience suggests that the scientific community will rapidly adjust. Similarly, how names are listed in databases is a matter of choice for their curators (although ICSP naturally encourages the use of correct names, as defined in the ICNP),” which points out that the community is free to use the previous names and proposed names per their own wishes. Such ambiguity in using different phylum names will add more confusion, as there is no uniform way to proceed.

Below, we have highlighted several concerns that have come to the fore given the newly proposed phylum names (6) based on the ICSP-sanctioned nomenclature framework (4, 5).

1. If Pseudomonas is the type genus for the phylum *Proteobacteria*, why is the ICNP guideline-based phylum name proposed as “*Pseudomonadota*” instead of “*Pseudomonasota*”? What is the justification for using “Pseudomonad” as a base type-genus word instead of “Pseudomonas”? This is a significant concern, as “Pseudomonad” is a generic word representing diverse species of the genus Pseudomonas and thus should not be considered a genus name in the current context. So why was the proper type genus prefix not used in this instance?
2. Along with “-ota,” the suffixes “-dota,” “-icota,” “-nota,” “-richota,” and “-tota” have been haphazardly appended to type genus names in 9 of 42 instances (6) (Table S1). “Actinomyce-ota” is arguably more syllabically convenient than “Actinomyce-tota.” Similarly, “Aquifex-ota” is more straightforward to pronounce than “Aquif-icota.” Overall, these nine inconsistent cases (Table S1, bold red highlight) will impede the uptake and acceptance of new phylum names.
3. Thus far, the ICSP has not discussed changing the names of class ranks, despite those too being rife with anomalies at both root word and suffix levels. Is the ICSP planning to harmonize name-altering rules changes for all taxa based on type genus root word and constant suffix?
4. *Proteobacteria* is the largest phylum in the *Bacteria* kingdom in terms of identified and studied organisms and has several classes that share the suffix “-proteobacteria”. Does the ICSP posit that the names of established classes Alphaproteobacteria, Betaproteobacteria, Deltaproteobacteria, Gammaproteobacteria, and Oligoflexia will be changed? For example, should “*Alphaproteobacteria*” instead be called “*Alphapseudomonadota*,” or “*Alphapseudomonadia*,” or something else using its respective type-genus name?
5. Previously, a clear difference (i.e., usage of the suffix “-archaeota”) existed between bacterial and archaeal phyla, as seen with established names such as *Crenarchaeota*, *Thaumarchaeota*, *Euryarchaeota*, etc. However, the proposed names (6) do not convey this distinction and might instead lend themselves to mix-ups with other bacterial phylum names. For example, changing *Thaumarchaeota* to *Nitrososphaerota* could lead to the latter being confused with the proposed bacterial phyla names *Nitrospirota* and *Nitrospinota* (6). To maintain this distinction, we recommend maintaining “-archaeota” as a suffix for phylum names belonging to the kingdom *Archaea*.
6. Despite *Euryarchaeota* having the highest representation in the kingdom *Archaea*, the phylum name was not changed (6) to *Methanobacteriota*, based on its type-genus Methanobacterium. We do not understand the reason behind this.
7. Using the type genus name may be advantageous in certain instances; however, the genus with the largest number of sequenced genomes can also be considered an option in today’s modern genomic era. In many instances, a type genus in each phylum has not been extensively sequenced/studied compared to other genera. For example, Actinomyces (i.e., the type genus within the phylum Actinobacteria), has only 285 sequenced genomes and 8,273 associated publications, whereas genera such as Mycobacterium, Streptomyces, and Bifidobacterium have 8,027, 2,537, and 1,706 sequenced genomes and 128,729, 30,007, and 11,567 associated publications, respectively (Fig. 1 and Table S2). Similarly, within the phylum *Firmicutes*, the type genus Bacillus is represented by 5,801 sequenced genomes and 113,637 associated publications, whereas Streptococcus and Staphylococcus have >17,600 sequenced genomes each and 116,933 and 174,341 associated publications, respectively (Fig. 1 and Table S2). In this study, genome count searches were performed using the prokaryotes.txt file (downloaded on 20 January 2022 from the NCBI FTP page); publication searches were performed on 23 February 2022 using PubMed. Overall, this raises the question: is the foremost determined type genus a reliable representative for its respective taxon?
  10.1128/mbio.00970-22.2 TABLE S2 Representative data for the distribution shown in Fig. 1. Genus names in red are the type genera in their respective phyla. Download Table S2, XLSX file, 0.01 MB. Copyright © 2022 Panda et al. 2022 Panda et al. https://creativecommons.org/licenses/by/4.0/ This content is distributed under the terms of the Creative Commons Attribution 4.0 International license.
8. Nowadays, authors are free to name an identified organism or its respective taxon. While we agree with this principle, it can create considerable chaos, such as in the case of Myxococcus llanfairpwllgwyngyllgogerychwyrndrobwllllantysiliogogogochensis (named after the town in Wales where it was isolated) (12), a name which has been validly published under the ICNP (https://lpsn.dsmz.de/species/myxococcus-llanfairpwllgwyngyllgogerychwyrndrobwllllantysiliogogogochensis; accessed 5 April 2022). Where should we draw the line? Some regulations governing these names must be implemented. Instead of changing names after the fact to bring consistency, we suggest that nomenclature at any taxon level should be first proposed to the ICSP as a sort of quality control to verify that it satisfies the letter and spirit of the governing rules. Only then should it be validated and published.
9. We also unequivocally state that we are not criticizing the renaming/reclassification of new organisms/taxa based on new morphological, genomic, and/or phylogenetic information. On the contrary, the reclassification and renaming of different groups from previously known monophyletic groups must continue, provided that ample support exists from new data.

Principle 1.1 of the ICNP rulebook (4) is “Aim at stability of names.” Principle 1.2 goes further and exhorts to “Avoid or reject the use of names which may cause error or confusion.” We trust that these two foundational ICNP principles are sufficiently convincing to keep the same root word in the name of each of the six phyla described above that represent ~90% of mentions in today’s microbial taxonomy sphere of influence (Fig. 1). Additionally, the article proposing validated names for 42 phyla (6) states that “The Judicial Commission of the ICSP can make exceptions and conserve extensively used names of phyla formed in different ways.” Therefore, we firmly believe this to be the perfect juncture at which to follow these rules.

We firmly support using the “-ota” suffix for all phyla to provide uniformity; however, we suggest the usage of the established root word for these six phyla for avoiding unnecessary confusion: *Proteobacteria* (new name: *Proteobacteriota*), *Firmicutes* (new name: *Firmicuteota*), *Actinobacteria* (new name: *Actinobacteriota*), *Tenericutes* (new name: *Tenericuteota*), *Crenarchaeota* (same name: *Crenarchaeota*), *Thaumarchaeota* (same name: Thaumarchaeota), and *Euryarchaeota* (same name: *Euryarchaeota*). This would also follow principle 4 of the ICNP rule book (4): “The primary purpose of giving a name to a taxon is to supply a means of referring to it rather than to indicate the characters or the history of the taxon.”

Overall, our suggestions will help maintain consistency across all phylum names and allow current and future researchers to focus on the science itself instead of getting bogged down by unnecessary nomenclature confusion while analyzing data, reading previous literature, and communicating microbiology. In words attributed to the industrialist and visionary Henry Ford, “We do not make changes for the sake of making them, but we never fail to make a change once it is demonstrated that the new way is better than the old way.”

### Data availability.

Data used in this analysis were procured from the NCBI public repository.
